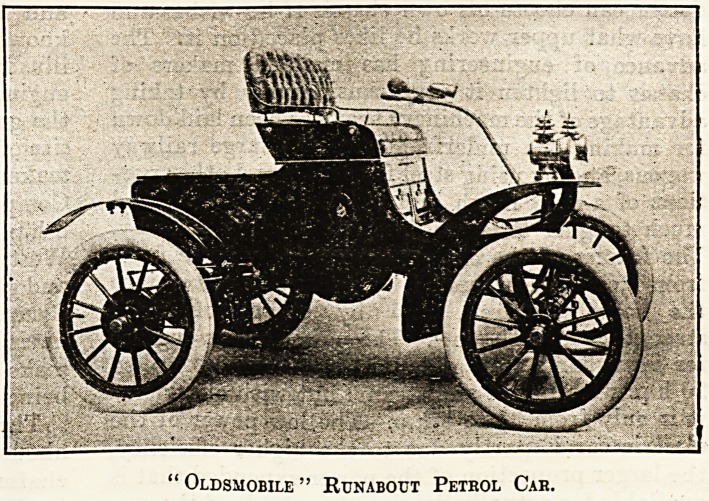# Motor Cars for Doctors

**Published:** 1906-05-12

**Authors:** 


### HOSPITAL ADMINISTRATION.

### CONSTRUCTION AND ECONOMICS.

### MOTOR CARS FOR DOCTORS.

#### CARS SUITABLE FOR PRACTITIONERS.

There are three principal forms of motor-cars suitable for the medical profession, two of which are made for petrol, steam, or electric driving. There is first the Runabout, a car very similar to the dog-cart, which can be fitted with a hood on the lines of the cape-cart, and with a back seat, and whose price runs at from £100 upwards, with additions for the cape-cart hood and back seat if required, and which has an engine of 6 to 8 h.p. It is at present only made in petrol. Then there is the brougham, of which so many are now seen about London, made to carry two, three, or four passengers, and made also to drive from a box-seat, as with the horse carriage, or from inside, a great improvement upon horse-drawn vehicles, as the doctor who is so inclined can drive himself, without any inconvenience or loss of dignity. The brougham is made in all three types, petrol, steam and electric. And there is an extension of the brougham, the landaulette, which is practically a long brougham. There are also variations of these, such as the open car with tonneau body, entering from the side, and capable of being converted into a closed vehicle at will. The tonneau is the special seating portion which has grown up with motor-cars, something like the governess cart. The building of motor-cars has followed the tendency to specialising that is one of the features of the time. The chassis, as it is called, the body of the car, that which carries the machinery, is built by engineering firms; while the upper portions, which belong to the coachbuilders' department, are built by coachbuilders, so that a doctor can choose his own chassis if he wishes and have what upper works he likes placed on it. The advance of engineering has enabled makers of chassis to lighten it very considerably by taking advantage of the machinery that has been laid down for making the underframes of the large railway wagons, and by using steel for shafts and other portions of a very much higher tensile strength, and much tougher than was possible in days gone by. The frames of the chassis are made by some firms from steel plates pressed by hydraulic power into the form required, and by others of wood strengthened with steel. The power of the engines for broughams and landaulettes is from 10 h.p. to 20 h.p. with petrol and steam; but with electricity it is only from 3½ to 5 h.p. The low power of the electric car is partly due to the higher efficiency, the larger proportion of the power expended that is available for actually driving the wheels of the car, owing to the direct connection of the motor with the axle. It is also partly due to the fact that at present the electric car is only made for short distances, 25 miles being the effective limit, and that it is not intended to do heavy work or to run at a high speed. The cost of the brougham and the landaulette run from £350 up to £700, according to the power of the engine and the fittings of the body of the car. The doctor who wishes to can spend as much as £1,200 or more on a car, which will be a very luxurious affair. The running costs of the various cars are claimed to be from ½d. per mile for some of the small petrol cars, up to 6d. per hour for the electric brougham or landaulette, which is merely for actual expenses while running. Threepence per mile appears to be a fair average cost, to include all charges, interest, and repairs, not including the wages of a chauffeur. The doctor would be well advised to have his own coachman trained to drive. All the makers of cars are quite willing to give coachmen practical instruction free of charge if a car is bought from them. There is nothing in the mechanism of any car that the average coachman, who puts his mind into it, cannot master; and in addition, he has the great advantage of knowing the vagaries of horses that he meets on the road. The doctor who has the time and the inclination will also do wisely to make himself as fully master of the details of his car as possible, and for the same reason that he makes himself master of each of the instruments he uses.

#### Some of the Cars on the Market.

The light Runabout car is made in various forms by several makers. It usually has a single engine of 6 to 7 h.p., though in a few cases it is made with 8 to 10 h.p. That sold by Messrs. Charles Jarrot and Letts, of Marlborough Street, London, W., known as the Oldsmobile, of which we give an illustration, is a good example. It has a 7-h.p. engine fixed horizontally under the body of the car, the gearing being epicyclic. Other makers of this size of car are: Messrs. Panhard and Levassor, who make an 8-h.p. landaulette; the De Dion Bouton Company, who make cars on the same lines with 8-h.p. and 9-h.p. engines; Messrs. Vauxhall and West, who make a car with three seats, a cape hood, and glass screen, with a 9-h.p. engine; the Speedwell Company, who make 6-h.p. and 9-h.p. cars, the latter having side entrances; the Wolseley Company, who make a 6-h.p. car with single cylinder, the engine being horizontal, and an 8-h.p. with two cylinders.

The Duryea Motor Company also make a care of about the same size, but more on the lines of the chaise. It is shown in two of the illustrations, one having a reversed seat like the dogcart. They also make larger vehicles for seating four and upwards. The Duryea Company use epicyclic gear on all their cars, but they claim that the gearing is very rarely in use, and therefore a large proportion of the engine power is nearly always available for driving, this meaning economy in every sense.

Of the broughams and landaulettes the makers are very numerous and the arrangements various.

Duryea "Runabout" Petrol Car.

Another Form of Duryea "Runabout."

Argylls' Petrol Landauette, Closed.

Argylls' Petrol Landauette, Open.

The engine power is from 10 h.p. to 20 h.p., and while two cylinders is the rule, three, and sometimes four, are fitted. The Argyll is a good example of the landaulette, and the Allday and Onions of the brougham, the latter being arranged to drive from the inside. Examples of the cars of both of these firms are shown, closed and open. The landaulette is made either without the upper works, simply with

Alldays and Onions' Petrol Broughan, Closed.
(Steered from Inside).

Alldays and Onions' Petrol Brougham, Open.

"Oldsmobile" Runabout Petrol Car.

a tonneau body, entering from the side, or with the usual carriage body, and to seat two, three, four, five, or occasionally six persons. Cars in this category made by other firms of these sizes are: A

20-h.p. landau, with four cylinders, by the Lanchester Company; a 20-h.p. double phaeton, with detachable top, seating six, by the Brotherhood-Crocker Motors, Limited; the "Allround" car, by Messrs. Maudslay, with three-cylinder engine, flat canopy, and glass wind-screen; Messrs. Panhard and Levassor, 15-h.p. and 24-h.p. engined cars; the Wolseley Company, 12-h.p., 15-h.p., and 18-h.p. cars; the Humber Company, 10 to 12 h.p. and 16 to 20 h.p. cars, with four cylinders, the latter with side entrance; the De Dion Bouton Company, 12-h.p. and 15-h.p. cars; the Speedwell Company, Messrs. Friswell, the Delaunay-Belleville Company, and others, 10 to 12, 16, and 18 to 24 h.p. cars; the Cras-Mercédès Company make a 20 to 25 h.p. coupé landaulette.

Of the steam cars, the White is the best known, and has been deservedly successful. Its steam generator is carried in the fore part of the car, and consists of a coil of tubes, into the top of which the water enters, the petrol burner being below, and the steam being thoroughly dried, and what engineers call superheated, before it enters the engine. When steam is generated there is always some water carried over with the steam, in a finely divided state, which condenses on the first slightly cooler body it meets, and gives trouble, as it absorbs heat from some other part to become steam again. By superheating, as in the White steam generator, this is prevented. The engine is of the compound type, the steam passing through the two cylinders, which are vertical, in succession. It carries sufficient petrol or benzine to run 150 miles. The water is sufficient for the same distance, it being, as explained, used over and over again, the condensed steam returning to the water-tank. In the Turner-Miesse steam car there are three cylinders fixed horizontally under the car, the drive to the wheels being by sprocket wheels and chains. The car runs with paraffin, a small quantity of petrol being required to start it, and it carries a supply for 100 miles run. The steam generator is on what is called the "flash" principle. It consists of tubes, but these are made hot, and water is then admitted to them in a small quantity, instantly becoming steam. The S.M. car is something on the lines of the Turner-Miesse. It has a flash boiler, and burns paraffin, water and paraffin being carried sufficient for 150 miles. All steam cars are worked with what engineers call a slide valve, one which slides over the ports by which steam is admitted to the cylinder, connecting each in turn with the steam supply and with the exhaust. They also all work with what is called link motion, by which the direction of motion is reversed by reversing the entrance of the steam to the cylinders. All have also some form of control of the supply of fuel by the steam pressure, so that when the car is standing a dangerous pressure may not be generated.

In the early days of electric cars a very pretty form of Runabout was made, but it does not appear to be on the market now—only broughams anl landaulettes. The company who have developed this branch of motoring the most are the City and Surburban Electric Carriage Company, whose headquarters are at Niagara, close to St. James's Park Station on the Underground Railway, London, S.W. They arrange to sell a car outright, but afterwards to relieve the owner of all trouble in connection with it. They garage it for him, see to the charging of the batteries and all repairs, and he has it at call at all times either by telephoning or at the times arranged for. This unfortunately only applies at present to London within reach of Niagara, but there is no reason that similar arrangements should not be made in the large provincial towns. The extra cost of running electric cars is often not important considering the convenience of the whole arrangement.

## Figures and Tables

**Figure f1:**
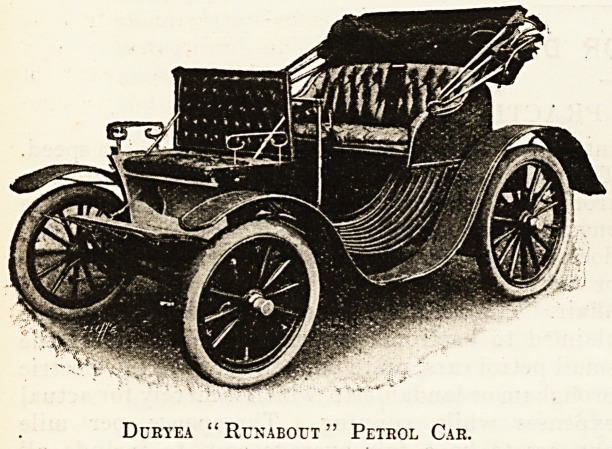


**Figure f2:**
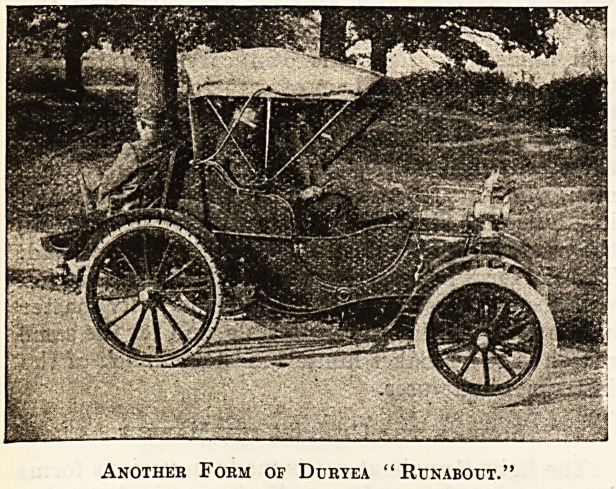


**Figure f3:**
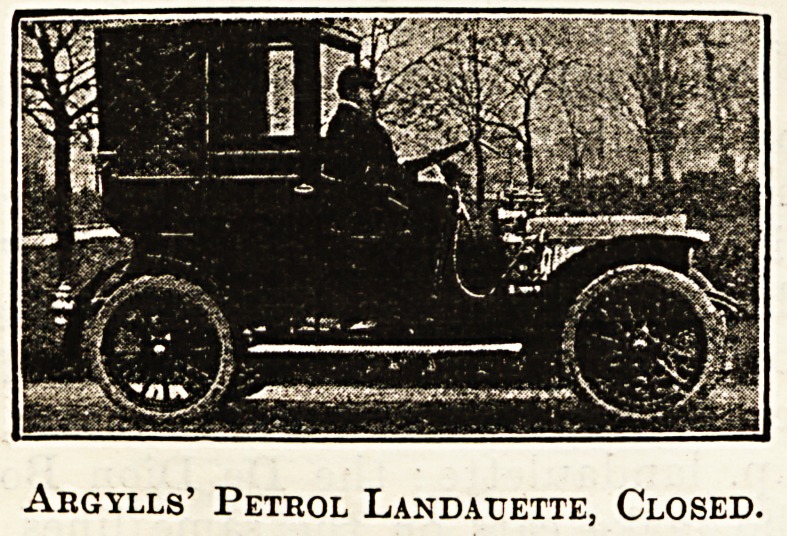


**Figure f4:**
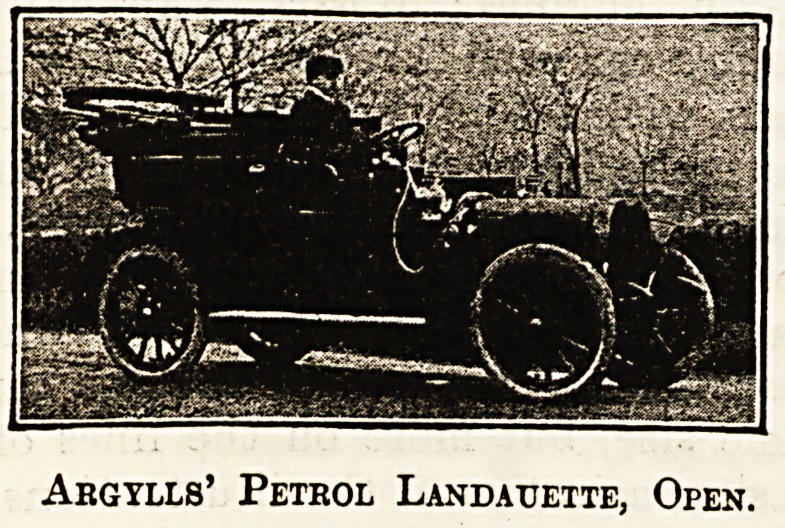


**Figure f5:**
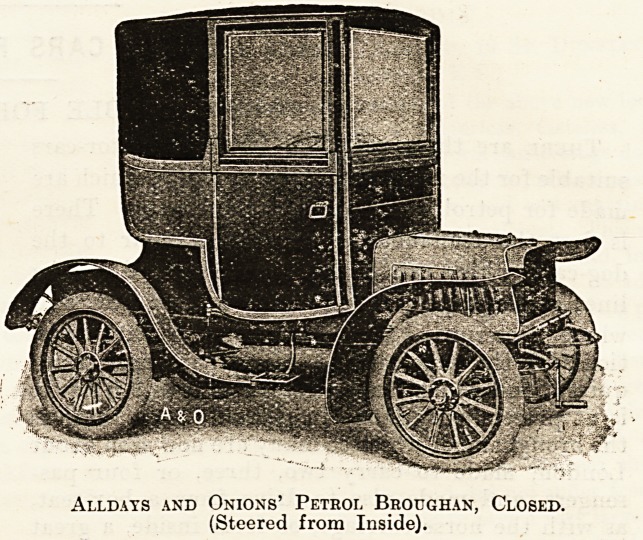


**Figure f6:**
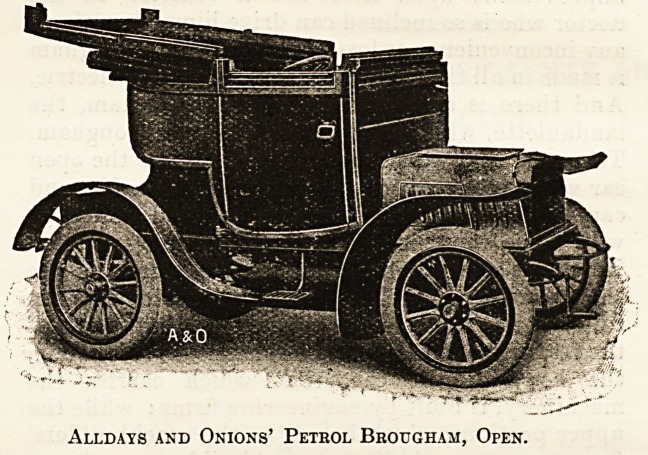


**Figure f7:**